# The Provision of Social Support in an Online Support Forum for Caregivers of People With Comorbid Dementia and Cancer: Content Analysis Study

**DOI:** 10.2196/72217

**Published:** 2025-09-29

**Authors:** Mollie Louise Price, Claire Surr, Brendan Gough, David Howe, Laura Ashley

**Affiliations:** 1 School of Humanities & Social Sciences Leeds Beckett University Leeds United Kingdom; 2 Centre for Dementia Research, School of Health Leeds Beckett University Leeds United Kingdom

**Keywords:** internet-based intervention, online health communities, peer support, social support, support groups, content analysis, caregiving, carers, multimorbidity, cancer, dementia

## Abstract

**Background:**

A growing number of people are living with comorbid dementia and cancer (CDC), and they are particularly likely to require support from family caregivers. Carers of people with CDC play a vital supportive role but have reported unmet support needs, including a lack of CDC-specific information resources and peer support. A targeted online peer support forum may provide an accessible way to help address unmet needs of carers of people with CDC.

**Objective:**

This study aimed to explore the types and frequency of social support provided on an online peer support forum for caregivers of people with CDC, hosted by a dementia charity in the United Kingdom.

**Methods:**

We conducted a mixed methods study using descriptive statistics and qualitative content analysis. All posts (N=893) on the forum since its launch in November 2018 to April 2024 were exported into Microsoft Excel for analysis. Descriptive statistics were used to examine forum use and user characteristics. Deductive content analysis was conducted to explore the types and frequency of social support provided on the forum. Posts were analyzed according to an adapted version of Cutrona and Suhr’s Social Support Behavior Code, consisting of 5 main categories of support: informational, emotional, esteem, network, and tangible. Coding was completed independently by 2 coders, and any coding disagreements were resolved by reaching a consensus through discussion.

**Results:**

A total of 258 usernames posted on the forum since its inception. There were 893 posts; 583 (65.3%) were coded as providing social support. All 5 Social Support Behavior Code categories were present in the forum posts. Informational support was the most common type of social support provided on the forum, which mostly involved providing suggestions for caregiving and coping strategies and sharing personal experiences that provide CDC-specific knowledge or insight. This was followed by emotional support, which consisted mostly of expressing shared understanding and empathy for caregivers in their unique situation of CDC and providing expressions of care for the recipient’s well-being. Esteem, network, and tangible support were less common, though they included providing validation and relief of blame to other caregivers, typically in decision-making regarding cancer treatment; reminding caregivers that others were available on the forum for support; and expressing willingness to answer questions about their CDC caregiving experience.

**Conclusions:**

This study demonstrates the use and value of a CDC-specific online forum as a source of social support for carers of people with CDC, facilitating users’ access to CDC-specific information and peer support. The relatively new forum shows promise as a free and accessible resource that can contribute to addressing carers’ informational and peer support needs.

## Introduction

### Background

Prevalence of both cancer and dementia increases with age [1,2], and owing to the aging population and rise in multimorbidity, a growing number of people are now living with comorbid dementia and cancer (CDC) [3-5]. A recent analysis of UK primary care data found that 1 in 13 people aged ≥75 years with cancer also have dementia [3], though this is likely to be an underestimation due to dementia underdiagnosis [6]. In recent years, there has been a surge in research examining cancer care and outcomes for people with CDC [7], and this work has consistently highlighted the crucial role of family carers for this group [8-11]. Dementia is characterized by progressive cognitive, functional, and behavioral impacts, such as impairments in memory, comprehension, language, and judgment [12]. Therefore, people with CDC may have difficulty with recognizing and communicating cancer-related symptoms, making treatment decisions, navigating cancer care and treatment, and self-managing symptoms and side effects [8,10,13-15]. Thus, people with CDC are especially likely to require support from family caregivers, who are integral in enabling people with dementia to access and undergo cancer treatment and care [8,10].

The adverse impact of caregiver burden on physical health outcomes and psychological well-being is well-documented [16-18], and providing care for people with *multiple* chronic conditions, especially when dementia is present, is particularly burdensome [19,20]. Recent qualitative work has documented the complex challenges faced by carers of people with CDC. For instance, the combination of extensive care responsibilities required for cancer management and the negative impact of dementia on care recipients’ ability to understand and self-manage such care results in heightened responsibility for carers, who report intense feelings of pressure to act as their care recipient’s advocate [21]. Carers play a crucial role in the recognition and management of cancer symptoms and side effects, helping care recipients to cooperate with cancer investigations and treatment and assisting with difficult cancer-related medical and nursing care (eg, ostomy or wound care), all of which can be further complicated by behavioral and psychological symptoms of dementia [10,21,22]. Dementia also produces greater complexities in cancer treatment decision-making, and where care recipients lack capacity, family carers report ethical dilemmas in deciding whether to pursue anticancer treatment or a palliative care pathway and associated feelings of guilt and perceived stigma around their decision-making [13,21-23]. Carers of people with CDC report feelings of uncertainty, anxiety, stress, and guilt [10,21], and where care recipients have a reduced insight into their cancer diagnosis and prognosis, carers report feeling as though they are carrying the emotional burden alone, contributing to feelings of loneliness and isolation [21,22].

Carer supportive interventions and services are imperative to reduce the negative impact of caregiving on carers’ own health and well-being, and several studies have highlighted the need for support services and resources for caregivers of people with CDC [9,10,23]. Our team’s previous in-depth qualitative study was the first to examine the psychosocial support needs and preferences of caregivers of people with CDC [21]. The participants voiced that the practical and emotional burden of their caregiving role was exacerbated by a lack of CDC-specific information and resources to help them navigate their dementia caregiving role in the context of comorbid cancer, which left them fraught with uncertainty. Carers of people with CDC voiced a desire for peer support opportunities, in particular for CDC-specific information and emotional support from others who understand the unique challenges of navigating cancer-related decision-making, treatment, and care for people who are also living with dementia. Despite expressing a need for support from caregiving peers, none of the interviewed participants had met other carers of people with CDC, and owing to their intense care regimes, they highlighted the difficulty of attending face-to-face support groups due to lack of time and difficulty leaving their care recipient. In response to these findings, which underscored the need for tailored CDC-specific peer support and information for this group, our team established a new online peer support forum to help connect carers of people with CDC and address these unmet support and information needs. Subsequent qualitative studies involving carers of people with CDC, by our team and others [7], have also corroboratively highlighted a need and desire for CDC-tailored information and support among this group.

According to the stress process model of caregiving, access to social support, such as that from peers, has the potential to improve an individual’s appraisal of a stressor and buffer the impact of caregiving stress on carers’ well-being [24,25]. Social support is a broad concept and can be categorized into different types of supportive behaviors. Several classifications have been proposed; these most commonly include informational support (eg, provides knowledge, advice, or factual input), emotional support (eg, provides empathy, encouragement, or expressions of care), and instrumental support (eg, offers to provide physical services, goods, or aid) [26,27]. Peers facing similar stressors are thought to be in a unique position of understanding to offer specific social support that cannot be provided by those in an individual’s existing networks [28]. Peer support has been associated with positive outcomes in caregiver samples, including greater perceived social support, confidence, and coping, and lower levels of depression and anxiety [29-31]. Moreover, as peer support groups are based on fostering mutual aid [32], participants often also report deriving benefit from providing support to others, such as an increased sense of confidence, self-efficacy, and achievement [32-35]. Indeed, carers of people with CDC, particularly those no longer in their caring role, reflect on having acquired specialized knowledge and expertise over their caregiving trajectory and having a desire to help others in the same position as a way of finding positive meaning from their experience [21].

While peer support group interventions have traditionally been delivered face-to-face, the now ubiquitous use of computers, mobile devices and social media has provided an accessible alternative means of support provision. Increasing numbers of people now use online support groups to seek and provide information, advice, and support [36], and greater caregiving demands and perceived burden have been found to predict greater likelihood of visiting online support groups [37]. Content analyses of online peer support groups for other communities show that, similar to face-to-face support groups, they can provide an important source of social support, most commonly informational and emotional support [38-47]. Correlational studies have found positive associations between support received from online groups and users’ psychological well-being, including lower levels of depression and loneliness and increased quality of life, optimism, and empowerment [48-52], and use of online support forums has been found to buffer the effect of caregiver strain on mental and physical well-being [53].

Online peer support communities have several unique features that may make such support particularly suitable for carers of people with CDC. First, they can typically be accessed 24/7, from home, and do not require the simultaneous presence of group members, therefore overcoming key barriers to attending face-to-face support groups reported by carers of people with CDC [21,54]. The asynchronous nature of online support forums removes the perceived obligation to respond, and users can be more selective in the information and support they obtain [55,56], which further allows carers of people with CDC to access such support around their busy caregiving regimes. Second, the ability to be anonymous and the absence of social cues in text-based communication helps create a nonthreatening environment in which carers may feel more able to discuss sensitive or stigmatized topics without fear of judgment [55,57]. Given the ethical dilemmas and sense of guilt and perceived stigma that carers of people with CDC can experience when supporting cancer treatment decision-making [21-23], an online support community could provide a “safe space” for carers to express such feelings with understanding others. Finally, online support groups remove geographic barriers and can thus bring together more widely dispersed and diverse groups of people, increasing possibilities for connectivity with peers and access to specialized information beyond that available in offline support networks [55,58-61]. Owing to the single-disease framework that still dominates health care (ie, care systems focused on treating single diseases while not appreciating multimorbidity), finding information and support relating to a specific combination of chronic conditions is difficult. An online peer support community could facilitate bringing carers of people with CDC together, providing a sense of community with similar others that they do not otherwise have. Furthermore, the collective experiences and knowledge shared in such a community could provide a valuable and specialized information resource specific to the combination of dementia and cancer, helping to address support and information needs unmet in their offline support networks [60].

### Objectives

An online peer support community may thus provide an accessible way to help address the support needs of carers of people with CDC. In partnership with UK dementia charity Alzheimer’s Society, in 2018 we established an online support forum specifically for carers of people with CDC, which, to our knowledge, is the first of its kind specifically for this group [62]. In this paper we examine, for the first time, the types and frequency of social support provided on this CDC peer support forum since its inception.

## Methods

### Launching the Online Forum for Carers of People With CDC

Our team applied for and secured a research partnership with the Alzheimer’s Society, proposing a new designated peer support forum specifically for caregivers of people with CDC to be created by the charity and housed within their existing online community: Dementia Support Forum (DSF). DSF is a free online community for people with dementia and their carers and supporters. It houses a number of different forums for subcommunities of people affected by dementia (eg, “I have dementia” and “I care for a person with dementia”). Within each of the forums, users can create or respond to discussion threads to both seek and provide information and support, such as asking and responding to questions, sharing experiences and advice, expressing concerns, and connecting with other users. DSF forums and discussion threads can be viewed publicly by anyone who visits the website. However, to post within forums or to privately message users, individuals must register as a member, which is cost-free. Although the forum is public, the online community guidance pages advise users on how to protect their privacy online, such as discouraging the use of identifiable information in their usernames or posts. In addition, the exchange of personal contact information is not permitted on the online community. The community is managed and moderated by a small team of trained staff and volunteers, who monitor user posts daily. In the context of health-related online communities, such moderation is important to reduce the risk of misinformation and to safeguard users’ well-being, maintaining a safe and supportive environment [63].

The new forum was titled “Caring for a person with dementia and cancer” and launched at the end of November 2018. It aimed to provide users with targeted CDC-specific information and peer support from others who understand the unique challenges of caring for a person with CDC. While the forum is hosted by the Alzheimer’s Society, we have worked in partnership with the charity to contribute to the content and activities on the forum. For instance, to promote and facilitate initial discussion and use of the new forum, we implemented several launch activities. These included (1) advertising the launch via the Alzheimer’s Society’s monthly newsletter, social media channels, and within the DSF online community; (2) populating the forum with some existing discussion threads concerning caring for a person with CDC that had previously been posted in other areas of DSF; (3) creating some initial discussion threads based around specific topics relevant to CDC; and (4) hosting 2 expert question and answer (Q&A) threads on the forum with a Macmillan dementia nurse (a nurse specializing in providing care and support to individuals affected by both dementia and cancer, funded by UK charity Macmillan Cancer Support). Such activities are common when launching new online support forums [45,64].

### Data Collection

All user posts on the forum since its launch in November 2018 were extracted into Microsoft Excel for content analysis on April 14, 2024, with the exception of the expert Q&A threads (as these were regarded as specialist rather than peer support). To protect user anonymity, potentially identifiable information (eg, names of people and places) was removed. “Posts” is used to describe both threads and replies. Descriptive data were also extracted into a data-charting form in Excel, including number of thread views and replies, number of individual posters, and, where provided on their public user profile, sociodemographic characteristics of posters (ie, gender, age, and caring status).

### Data Analysis

Descriptive statistics examined the frequency of forum use and characteristics of forum users. Qualitative content analysis was used to examine the types and frequency of social support exchanged on the forum. Deductive content analysis, situated within a postpositivist research paradigm, involves coding data according to preexisting coding categories that are imposed by previous theory or research findings; this is the process of “deduction” [65,66]. The aim of the content analysis in this study was to examine the provision of different types of social support on the online forum. A deductive approach was thus taken to categorize the data according to an existing theoretical framework of social support: the Social Support Behavior Code (SSBC) [27]. The SSBC is a coding framework originally designed to categorize social support communication behaviors during stressful life events and comprises 23 behaviors within five main categories of social support: (1) informational, (2) emotional, (3) esteem, (4) network, and (5) tangible assistance. Since its development, the SSBC has been adapted for suitability to an online context and used widely to explore the social support behaviors exchanged in online peer support forums and groups for a wide range of samples experiencing different health conditions, or families and carers affected by these. These include, for example, people with depression [39], HIV or AIDS [45], Huntington disease [42], irritable bowel syndrome [41], patients who underwent bariatric surgery [38], people affected by methamphetamine [44], parents of people with autism spectrum disorder [47], families affected by childhood cancer [43], and carers of people with dementia [67].

The SSBC used in this study was adapted in line with other studies using the SSBC to analyze data from online support groups [38,40,42-45,47], preserving the core categories of social support but modifying specific subcategories for online relevance. For instance, some subcategories were removed due to lack of relevance to the remote or public nature of the community (ie, “listening,” “loan,” and “confidentiality”) [38,42,47]. Other subcategories and their definitions were modified for the online context (eg, “physical affection” was modified to “virtual affection”) [38,42,45,47], and lastly, new subcategories were added to reflect other types of social support common in online forums, such as “personal experience,” “community companionship,” and “expressions of care” [45,47]. The final coding taxonomy comprised 20 subcategories; refer to Table 1 for all the categories and their definitions.

Consistent with other studies using the SSBC [38,42-45,47], the framework was used to code for the provision of social support in individual forum posts (as opposed to analyzing dialogue between users, which was beyond the scope of this study). Posts could be coded as more than one type of support. All posts were coded independently by 2 postdoctoral researchers: MLP, who has experience in conducting qualitative research with carers of people with CDC, and DH, who has experience in psychosocial dementia-related research, including the provision of web-based support. Both authors coded a sample (134/893, 15%) and met to discuss and clarify understandings of the categories before going on to code all posts. On completion of coding, agreement between coders, calculated on a post-basis, was 82.0% (732/893) of posts. The 18.0% (161/893) of posts for which there was initial disagreement were discussed between the coders to reach a consensus on coding; a third opinion was not required.

**Table 1:** Definitions of the social support behavior codes and their frequency on the online forum for carers of people with CDCa.

Support type			Purpose of communication		Instances, n (%)
**Informational support (n=615)**					
	Suggestion and advice	Provides ideas or suggestions for action and coping		227 (36.9)	
	Referral	Refers the recipient to a specific source, community resource, or website		136 (22.1)	
	Situational appraisal	Helps reassess or redefine the situation being faced by the recipient, often in a way that makes it more positive or shows new information that could be helpful		37 (6)	
	Teaching	Provides detailed information, facts, or news about the situation or about skills needed to deal with the situation		58 (9.4)	
	Personal experience	Shares information about their own experience, often in a way that demonstrates specific knowledge		157 (25.5)	
**Emotional support (n=503)**					
	Virtual affection	Physical affection expressed (but virtually), such as “hugs and kisses”		59 (11.8)	
	Sympathy	Expresses sorrow or regret for the recipient’s situation or distress		110 (21.9)	
	Understanding and empathy	Expressions of understanding of the situation or disclosing a personal experience in a way that conveys understanding		174 (34.6)	
	Encouragement	Provides the recipient with hope, optimism, or confidence		43 (8.5)	
	Expressions of care	Conveys supporter’s engagement of recipient’s well-being		115 (22.9)	
	Prayer	Offers prayer or blessings for the recipient, reminders of faith		2 (0.4)	
**Esteem support (n=99)**					
	Compliments	Says positive things about the recipient or emphasizes the recipient’s abilities		21 (21.2)	
	Validation	Expresses agreement with the recipient’s perspective on the situation, emphasizes similar views		46 (46.5)	
	Relief of blame	Tries to alleviate any feelings of guilt the recipient has about the situation		32 (32.3)	
**Network support (n=207)**					
	Access	Messages that appeared to broaden the recipient’s social network by establishing access to a new member: “welcome” messages		112 (54.1)	
	Presence	Offers to be here, reminders that others are available to offer support		41 (19.8)	
	Community companionship	Indicates the community’s unique position to share experiences, the importance of community closeness, and gratitude for community support		54 (26.1)	
**Tangible assistance (n=7)**					
	Performs a task	Performs an action on behalf of the recipient or the group		5 (71.4)	
	Active participation	Offers to join the recipient in an activity; includes concrete plans or planning to do something together		0 (0)	
	Express willingness	Offers or expressions of willingness to help		2 (28.6)	

^a^CDC: comorbid dementia and cancer.

### Ethical Considerations

This study received ethics approval from the Psychology Local Research Ethics Committee at Leeds Beckett University (0223/MPMW). The DSF online community does not require account registration to view forum posts, and the guidance pages remind users that the community is publicly visible to anyone on the internet. It therefore meets the criteria for being perceived as public by its users and consent was not required for this analysis [68,69]. Such distinctions are typically used in other internet-mediated research studies to determine whether consent is required [41,44,70]. Nevertheless, permission was obtained from the DSF online community manager and all posts were anonymized during data extraction.

## Results

### Forum Use and Characteristics of Users

Since its launch in November 2018 (up until April 2024), 258 unique usernames posted on the CDC forum, with the number of posts by each user ranging from 1 to 58 (mean 3.50; SD = 6.30, median 2 (IQR 1-4)). Of the 258 users that posted on the forum, 63 (24.4%) provided their gender on their profile. Of these 63 posters, 49 (78%) were female and 14 (22%) were male. Most (242/258, 93.8%) identified themselves as current or former carers. Though it was not always clear if their care recipient had CDC or dementia only, 28.5% (69/242) specifically identified themselves as existing (or previous) carers of a person living with CDC; a further 4.1% (10/242) expressed that they were caring for someone with suspected CDC (eg, some posters were caring for a relative with dementia and were debating pursuing investigations and treatment for suspected cancer), and 1 user was a person living with CDC.

Since its launch, 893 posts have been made by forum users, including 110 discussion threads and 783 replies, with threads receiving an average of 7.13 replies (median 5 (IQR 3-10), range 0-34). Examination of viewing figures suggests a much larger number of people are using the forum simply to view the discussion between other users, and the 2 expert Q&A threads, rather than to post contributions themselves. As the website’s view counter rounds to the nearest thousand, we were unable to calculate precise viewing metrics, but views of the threads ranged from 388 to >15,000, with most threads having >2000 views (69/112, 61.6%). The 3 most highly viewed threads received over 10,000, 12,000, and 15,000 views, respectively.

### Provision of Social Support on the Forum

#### Overview

Of the 893 posts made by forum users, 583 (65.3%) were coded as containing aspects of social support. All categories of the SSBC were present in the forum posts, and out of the 20 SSBC subcategories, 19 (95%) were present. Some posts contained multiple subcategories of one type of support (eg, a post may contain both the “referral” and “situational appraisal” subcategories of informational support). Thus, the frequency of individual instances of the 5 main types of social support is different from the number of posts that contained each type of social support; both are reported throughout the findings. Table 1 presents the frequencies of all the SSBC categories exchanged on the forum.

#### Informational Support

Informational support was the most common type of support shared by forum users (n=615), and 393 (67.4%) of the total support posts from users contained at least one subcategory of informational support. “Suggestion and advice” was the most common type of informational support shared on the forum (n=227), followed by “personal experience” (n=157), “referral” (n=136), “teaching” (n=58), and “situational appraisal” (n=37).

“Suggestion and advice” included posts that suggested ideas or techniques to help others with coping or with their caregiving role. Such suggestions included appropriate support that could be accessed, questions to ask the physician, and techniques that may help improve difficult situations for carers of people with CDC (eg, not repeatedly reminding care recipients of their cancer diagnosis to reduce associated distress). For instance, one user shared their concerns about an upcoming visit from a Macmillan nurse potentially causing confusion for their mother with dementia and secondary liver cancer, and the difficulty of then having to reinform their mother of her cancer diagnosis. Another user replied to suggest asking the Macmillan nurse to not mention the cancer diagnosis in their visit:

I wonder if it would be possible for the Macmillan nurse to be vague with your mum as to why she is visiting. Perhaps she could say it’s just a check-up as she’s feeling so unwell and not mention the cancer. I imagine these nurses must have dealt with people with dementia before and I don’t see the point of upsetting your mum when she’ll forget what she’s upset about and I can’t see it making any difference to her treatment.

Some users provided suggestions and advice to others to help support cancer diagnosis and treatment decisions for a person living with dementia. For instance, one user posted that their mother had advanced dementia and was recently diagnosed with breast cancer; they expressed uncertainty over the decision for their mother to have a mastectomy and shared that they felt overwhelmed about making the “right decision.” Others replied offering advice to help with the decision-making process:

I think if I could, I would ask for the least intrusive treatment to be provided to ensure my mother could be kept as comfortable and pain free as possible.

I think that I would be asking what other treatment will be necessary along with the mastectomy e.g. chemotherapy, radiotherapy and considering whether your mother could cope with both the treatment and the side effects.

“Personal experience” included posts where users shared their own experiences of CDC or caregiving which provided knowledge and insight for others. For example, some users shared their experiences of making cancer care and treatment decisions, to help those currently struggling to make such decisions:

Dads oncologist said that she thought dad would not cope well with chemo and because of his dementia and frailty the gastro doctor would not consider the operation. We took their advice. Dad had palliative care only with just a stent to enable him to eat. Good decision as he is still here 10 months later having had a good quality of life.

When we had a decision to make about treatment, we opted for palliative care—but I have to say, I was very disappointed with what that consisted of and Mum’s last year with us was pretty appalling. Had I had all the facts at that time, I think I would have risked an operation—it couldn’t have made things worse for her—but refused chemo, which could have made her very sick on top of everything else!

Another user posted that they were concerned about their mother’s lack of communication and expressed that they did not know if it was due to her dementia progression or her lung cancer causing pain. One user shared their own experience of their mother who had dementia only:

Just to say, though, re not knowing you, with my mother (who had no other illnesses) this happened quite suddenly. One week her eyes would light up when she saw me coming—the next, they were just blank. After that I was just a ‘nice lady’ who made her cups of tea and brought her chocolate. So it could well be a ‘normal for dementia’ thing.

“Referral” posts on the forum included signposting to information or support on websites, in books, and via other sources. Signposting included weblinks to factsheets and information to help with getting care needs assessments, finding local support, the later stages of dementia, power of attorney, and how to communicate compassionately with people living with dementia:

You might find this link helpful in knowing how to respond to your mum...

Other referrals recommended approaching more specific support sources to help with a concern or situation, which included referrals to social services, the general practitioner, Local Authority Adult Services, support lines, speech and language therapy teams, and NHS 111 (a free, 24-7 nonemergency medical helpline in England). For example, one poster expressed that they were struggling to cope with the cancer caregiving responsibilities of a complicated medication routine and daily trips to the hospital coupled with their husband’s dementia and no longer wanted to be responsible for him. Several users responded directing the poster to call NHS 111 for urgent support:

I would phone 111 today. He needs an urgent assessment by social services. They have to take care of him by Duty of Care.

Ring 111 now before you crack. You must do this for your own sake.

Instances of “teaching” support included sharing information about the symptoms and diagnoses of dementia, progression and stages of dementia, capacity to make decisions regarding cancer treatment, the impact that operations and cancer treatments can have on dementia symptomatology, delirium following hospital visits and treatments, and teaching about available support and how it can be accessed. Examples of such support are as follows:

I’d like to explain that Alzheimer’s is in fact a form of dementia—there are others, like vascular dementia, Lewy Bodies and Picks, but they are all types of dementia, so if your mum has been diagnosed with Alzheimer’s then this means she has dementia.

Unfortunately, operations and treatments, as well as being gruelling, can accelerate the decline of a person with dementia.

Posts containing “situational appraisal” support encourage reassessing a situation to view and feel about it more positively by providing new information and insight; such situations included, for example, the threat of potential cancer investigations and diagnoses, and feeling dissatisfied with planned cancer treatment or care. For instance, one user posted that their mother’s physician had suggested a computed tomography scan to confirm whether her breast cancer had spread to her brain, or whether her symptoms were due to her dementia. They expressed doubt about whether the possible benefits of this would outweigh the stress of attending the appointment. Others responded helping to redefine the situation more positively, highlighting that receiving a specific cancer diagnosis can provide access to more systems of support:

I agree with many others. Diagnosis of cancer will get you into a system of support, even if the decision is made not to treat. Diagnosis of dementia will get you nothing. It may also give you a time frame.

Another user posted that her mother had been diagnosed with breast cancer and expressed her frustrations that the consultant had suggested that due to her mother’s dementia, a treatment plan of hormone tablets would be more appropriate than surgery. One user responded highlighting the negative impact some treatments and operations can have on dementia, suggesting the treatment plan may have been made with her mother’s best interests in mind:

You’re right—it is both shocking and depressing, but the consultant may have good reasons for giving this treatment. Obviously I don’t know your mum or what stage she’s at but most people find that surgery/a hospital stay often makes dementia worse. Could your mum cope?

#### Emotional Support

Emotional support was the second most frequent type of support provided on the forum (n=503), with at least one type of emotional support coded in 337 (57.8%) of the total support posts. “Understanding and empathy” was the most frequent type of emotional support shared on the forum (n=174), followed by “expressions of care” (n=115), “sympathy” (n=110), “virtual affection” (n=59), and “encouragement” (n=43). Just 2 instances of “prayer” support were coded in forum user posts.

“Understanding and empathy” includes posts that expressed understanding of the recipient’s situation. Users often responded to posts by sharing their own similar experiences and challenges in a way that conveyed shared understanding, showing the recipient that they are not alone:

I have the same dilemmas as you, is dad not finishing his breakfast because his tumour has grown or is he just not hungry? Does he sleep all day because of the cancer or is it the dementia?

Sounds familiar! My mum had oesophageal cancer and vascular dementia. She did know she was ill, and with the first diagnosis of cancer told the doctors that she didn’t want anybody—not even her—to know what stage she was at, nor did she want any treatment. Then things went downhill with both the dementia and cancer. What a nightmare for all concerned!

Other times users empathized with the recipient’s emotions:

Two things in your post really stood out for me. The word “frazzled” is such an apt description, and how I feel much of the time, despite my mother being in residential care.... I find your comment about cancer interesting too, and one that I can so relate to.

I can fully relate to all your feelings.

“Expressions of care” describes posts that demonstrate care for the recipient’s well-being. Often, such posts were simply wishes of care and support to those who were particularly struggling:

I really hope you can find some help soon, you need to look after yourself in this situation.

Take care of yourself Xx

Other times “expressions of care” were provided after users had shared more positive news, such as medical results or the receipt of support. For instance, one user, whose husband had Alzheimer and vascular dementia and was in hospital with suspected kidney cancer, followed up with the news that the scans had shown no signs of cancer and that he had been discharged from hospital. Other users expressed care to the poster by sharing that they were pleased to hear this update:

that is such good news and now he and you can relax. glad he’s now settled

That’s good to hear @username and I’m glad that your husband is now out of hospital and back in his care home. It must be a huge relief for you.

Another user posted to share that their husband who had mixed dementia had now been diagnosed with liver cancer, and that although he had deteriorated rapidly, they were grateful for the medical support available. Others shared that they were pleased the poster was getting the support they needed:

Having the proper support certainly makes a massive difference and I’m glad that you are getting it. Take care

I’m sorry to hear of your husband’s diagnosis, but am so glad you are getting good support from the general practitioner and other services.

“Sympathy” was often expressed in response to individuals who described difficulty coping with their caregiving role or uncertainty surrounding dementia and cancer symptoms and prognoses:

What a worry for you, I’m so sorry...

I truly feel for you in your position.

Some posts expressed physical affection virtually; this “virtual affection” was often shared in response to users who had expressed difficulty coping with their current situation:

I tearfully and gratefully accept your hugs and return the same.

Sending love and hugs your way. I know it’s not easy I really do x

“Encouragement” described posts that aimed to bolster recipients’ confidence and optimism, with such posts tending to encourage the recipient to “keep going”:

We are all with you and all desperately looking for an end and closure. Keep plodding with the rest of us.

You’re right, it will be hard, but you will get through it. When I moved my mother it was three months of hell (for me, not her—she knew nothing about it!) Stay strong

#### Esteem Support

There were 99 instances of esteem support in the posts analyzed on the forum. Of the total number of posts containing social support (n=583), 76 (13%) contained one or more instances of esteem support. “Validation” was the most common type of esteem support shared on the forum (n=46), followed by “relief of blame” (n=32) and “compliments” (n=21).

“Validation” described posts that expressed agreement with the recipient’s perspective or shared similar views. Many validation posts expressed agreement with forum users’ care or treatment decisions or decisions not to tell the care recipient about their cancer diagnosis:

I think you are right we have to filter out any information that is going to cause our loved ones any distress.... I think it’s the right thing to do, go with your instincts x

Others validated the recipient’s emotional response to a situation. For example, one user expressed that they had felt a sense of relief after their father’s cancer diagnosis. Another user replied to express their agreement:

Although it was a shock finding out mum has terminal lung cancer as well as Alzheimer’s, I agree it will at least bring it all to a end and we may be spared how our dad died of Vascular Dementia.

Another user shared her frustration that her friends did not understand the impact caregiving was having on her life and had told her that she should feel lucky she still has her mum. A user replied agreeing with her frustration and perspective on the situation:

What a load of rubbish telling you you’re lucky to have your mum, if she’s anything like my mum it’s not your mum! I think I’d have snapped

“Relief of blame” describes posts that try to alleviate any feelings of guilt or blame that are experienced by the recipient. Such posts typically attempted to alleviate feelings of guilt or shame in relation to cancer treatment decisions and associated complex emotions experienced by caregivers.

For example, one user posted about their father with Alzheimer disease being diagnosed with an aggressive form of cancer and how they were struggling with the idea of their father not having any surgery. They reported feeling “the burden of responsibility of doing the best for him.” One user who had also cared for their father with CDC and opted for palliative care responded to help alleviate feelings of guilt:

I don’t feel guilty and neither should you. These are difficult decisions to make and we do our best.

In another example, one user who was struggling caring for their mother with dementia and cancer expressed that they wished their mother would pass away from her lung cancer, to give them an “end to it all, a way out.” Another user replied trying to ease any guilt the recipient may have in feeling this way by assuring them that it was normal:

Don’t beat yourself up it’s natural to want it all to be over watching a loved one in so much distress has you praying for death so they can finally rest in peace.

“Compliments” describes posts that say positive things about the recipient or emphasize their abilities. Such posts on the forum were often in response to users who expressed doubt in themselves or their caregiving ability, such as expressing that they felt they were not doing enough or were letting their loved one down. Other users often responded to praise their caregiving efforts:

He was given every possible care by you both when he needed it most, you both could not have done anymore

It sounds to me like you are trying to do the best you can for your Mum, asking for med review, testing for urinary tract infections etc, it must be especially hard from a distance. Just because you cannot give your Mum the calmness and serenity you wish for her, does not mean you are letting her down

#### Network Support

There were 207 occurrences of network support shared on the forum. Of the total number of posts containing social support (n=583), 134 (23%) contained one or more instances of network support. Posts sharing network support included instances of “access” (n=112), “community companionship” (n=54), and “presence” (n=41) support.

“Access” describes posts that broaden the recipient’s social network by welcoming them to the community or helping to establish access to other members. Posts coded as “access” support on the forum mostly consisted of welcome posts: “Hello @username, a warm welcome from me.” Though some posts helped users to access other online community members caring for people with dementia and cancer, instructing them of how they could be notified when there are new posts in the CDC-specific forum:

It may be helpful to click on this link >watch forum< then you will be notified when there are any new posts and discussions in the dementia and cancer sub-forum that you may be interested in reading :)

“Community companionship” describes posts that emphasize the online community’s unique position to share experiences, the importance of community closeness or gratitude for community support. Community companionship on the forum mostly consisted of posts emphasizing the knowledge and support available on the forum and encouraging the recipient to keep posting:

Now that you have found us I hope you will keep posting as the membership has vast collective knowledge and experience.

I hope you find the forum to be a friendly and supportive place. Unfortunately what you describe is very common but you have come to the right place for understanding and support.

“Presence” describes posts that remind the recipient that there are others in the online community that are there for them, and are available to offer support:

Sorry I am not a lot of support but you are not on your own

We are all with you

#### Tangible Assistance

Of the total number of posts containing social support (n=583), 7 (1%) contained instances of tangible assistance support. Posts sharing tangible assistance included instances of “performs a task” (n=5) and “express willingness” (n=2). There were no instances of “active participation.” Tasks performed included looking up specific information for the recipient, such as the opening hours for an Admiral Nurse Helpline (a UK-based helpline offering specialist dementia support from dementia nurses), or venues in the recipient’s local area that cater for people with dementia:

I just had a look at their opening hours, they are open tomorrow—

Monday to Friday 9am-9pm

Saturday and Sunday 9am-5pm

Open bank holidays (9am-5pm), except 25th December

I hope you find them helpful.

Both instances of “express willingness” were posts offering to answer questions about their experience of caring for someone with CDC:

if you want to ask me anything else please do ask, you can message me if you want to and I will be happy answer.

## Discussion

This study (1) described the launch of a new online peer support forum specifically for carers of people with CDC, the first support of its kind for this caregiver group; and (2) provided the first examination of the types and frequency of social support provided by carers of people with CDC on an online forum.

### The Forum Provides a Source of Social Support for Carers of People With CDC

The findings demonstrate that the forum provides a source of various different types of social support for carers of people with CDC and was modestly used by its users. Deductive content analysis demonstrated the types of supportive communication that were exhibited on the forum using a theoretical framework of social support (SSBC) [27]. All 5 types of social support on the framework were exhibited on the forum; the most common support provided was informational and emotional support, and the least common was tangible assistance. This is in line with analyses of other online peer support forums [38,39,41,43-45,47].

In terms of informational support, users most commonly shared their own personal experiences which provided knowledge and insight for others, including for example, sharing their decision-making and cancer treatment experiences to help those currently struggling with such decisions, which is a particular challenge for carers of people with CDC [21-23]. Sharing suggestions or advice for coping and referrals to specific sources of support were also common forms of informational support. Regarding emotional support, users most commonly shared understanding and empathy of others’ experiences, which is a key mechanism of peer support groups [28]. Given that carers of people with CDC report that individuals in their existing social networks do not always understand their unique challenges, and that dementia-only support groups are often insufficient due to the lack of specificity to CDC [21], provision of understanding and empathy from other carers in the same situation on the forum is likely to be particularly beneficial.

The forum therefore provides a source of various different types of social support for users and thus has the potential to address some of the unmet informational and emotional needs expressed by carers of people with CDC [21]. From a transactional stress and stress-buffering perspective, use of the forum may have the potential to increase carers’ perception of social support availability, improving their appraisals of their ability to cope with their caregiving role and in turn, have positive effects on their well-being [25,71,72]. However, future work is needed to evaluate the perceived benefits of the forum by obtaining user feedback.

Online forums are low cost to implement and can overcome barriers to attending face-to-face support groups, such as those reported by carers of people with CDC [21,54]; and can help to connect carers who may otherwise lack access to CDC-specific information and peer support in their offline networks [21,55,58-61]. However, as with any online community, there are some limitations to consider regarding accessibility and use. For instance, the forum will not be accessible for all carers of people with CDC, such as those without internet access or those with limited digital literacy. Furthermore, the potential for misinformation to be shared is a particular challenge in the context of health-related online communities [63]. Nevertheless, expert Q&As have been hosted on the forum to provide specialist CDC support, and the forum is moderated daily by trained staff and volunteers, helping to mitigate this risk. Finally, while the asynchronous nature of the online forum may be beneficial for carers of people with CDC, who report particularly intensive caring regimes [21,55,56], it may be less suitable for those seeking immediate responses or support.

### Potential to Facilitate Positive Meaning-Making

Interestingly, instances of situational appraisal were also observed on the forum, whereby users helped others to reassess their situation in a more positive light. The situational appraisal subcategory of the SSBC is akin to the concept of positive reappraisal, a meaning-focused coping strategy whereby an individual attempts to control the meaning of a stressor by searching for positive aspects of the experience [73]. Positive reappraisal has been highlighted as an important factor in coping for carers of people with CDC [21], likely owing to the chronic and progressive nature of cancer and dementia not amenable to direct coping efforts [73-75]. This is important because according to the stress process model of caregiving, caregiver outcomes are not the direct effect of objective aspects of caregiving burden but are mediated by caregivers’ appraisals of stress. Thus, the ability to reappraise their situation in a more positive light has the potential to reduce the impact of caregiving on health and well-being outcomes [24,76-83]. Indeed, many interventions aim to facilitate positive reappraisal coping strategies [84-87], including on an online peer support platform [88]. In a randomized controlled trial of a web-based peer support platform that provides support with cognitive reappraisal techniques for people with depression [88], those that had greater depressive symptoms and low use of reappraisal techniques at baseline benefited significantly more from the intervention than from an expressive writing condition. Moreover, the intervention had significant indirect effects on depression and on perseverative thinking, which were mediated by improvement in reappraisal. Peer interactions on online platforms may thus provide a mechanism through which positive reappraisal can be facilitated. While this study demonstrated instances of peers facilitating positive reappraisals occurring naturally on the forum, it may be beneficial to promote this in a more structured way in the future (eg, by creating structured discussion threads that include quotes of positive experiences and reappraisals shared by carers of people with CDC and that invite shared discussion from users).

Furthermore, the various types of supportive communication exhibited on the forum also demonstrates that forum users utilized the forum to provide support to others as well as receive support themselves. Carers of people with CDC, particularly those who are now bereaved, have reported a desire to find positive meaning from their experience by helping others, sharing the specialized knowledge they have acquired over the caregiving trajectory [21]. Research has found that providing support to others on a peer support platform is associated with better psychological outcomes, including decreased depressive symptoms and health concerns, and increased subjective happiness, psychological quality of life, personal satisfaction, and use of positive reappraisal [89-92]. The ability to provide help to similar others on the forum may thus also have the potential to facilitate positive meaning-making for carers of people with CDC.

### Strengths, Limitations, and Future Directions

Strengths of this study include the use of 2 independent coders and high intercoder agreement, as well as the inclusion of all posts on the forum as opposed to a subset. Another strength of the study is the use of a validated theoretical framework of social support, which has been consistently used in other similar analyses of online communities for other populations [27,38,40,42-45,47]. However, as the analysis was conducted according to preexisting social support categories, future studies may wish to take an inductive approach to analyzing the content of posts on the forum, which could provide insights that are missed in deductive analyses.

While the findings demonstrate the availability of many types of CDC-specific social support on the forum, and thus the *potential* for the forum to meet unmet informational and emotional needs expressed by carers of people with CDC [21], future work is needed to obtain feedback from forum users to evaluate the perceived value and usefulness of the forum and the ways in which it could be improved. Furthermore, owing to the anonymous nature of online forums, many posters did not provide demographic information on their public profiles; therefore we know little about the users of the forum. Any future work gaining feedback from forum users could thus obtain more sociodemographic data, which could give valuable insights into who the forum may be most useful for.

After a trial period of 12 months, the Alzheimer’s Society decided to continue hosting the new forum as a permanent feature of their online community, and it is now being signposted in the Macmillan Cancer Support booklet for carers of people with dementia and cancer [93]. While the number of posts and individual posters on the forum has been moderate, viewing figures suggest a much larger number of people are using the forum. This is common in online communities; the “90-9-1 rule” describes a pattern of participation in online social networking whereby approximately 1% of the users are daily or heavy users, 9% are infrequent users, and 90% do not interact with others, but participate only to observe others interacting, often referred to as “lurkers” [94,95]. “Lurkers” still report benefits from using online support groups, and evidence suggests that “lurkers” and “posters” do not differ significantly in many health-related psychosocial outcomes [96-100]. This suggests that the forum could be useful to a much larger audience than those who are actively posting. Indeed, discussion threads between other users and the expert Q&As held with experts in CDC can act as a lasting resource of information for users who view the forum, regardless of whether they post to seek out support directly. Given that 1 in 13 people aged >75 years with cancer also have a diagnosis of dementia [3], and this group have more comorbidity and are especially likely to rely on support from caregivers [8,10], the information and support on the forum is likely to be beneficial to a significant number of people.

### Conclusions

This study demonstrates the value of a new CDC-specific online forum as a source of social support for carers of people with CDC. The forum shows promise in facilitating users’ access to CDC-specific information and peer support, which they typically do not have access to offline. It also has the potential to facilitate positive meaning-making through peer-to-peer interactions and through using the forum to help other carers of people with CDC. It offers a practically feasible and low cost method of addressing both informational and peer support needs expressed by carers of people with CDC and removes their reported barriers to accessing traditional peer support. In the current economic climate, e-resources like online forums are becoming more important sources of support than ever before and given that they are accessible and generally low cost to implement, the new forum provides a worthwhile contribution of support for this caregiver group.

## Abbreviations

CDC: comorbid dementia and cancer
DSF: Dementia Support Forum
Q&A: question and answer
SSBC: Social Support Behavior Code

## Data Availability

The dataset analyzed during this study may be available from the corresponding author on reasonable request.
